# Toward Inclusiveness and Thoroughness: A Paradigm Shift from More-ever-omics to Holovivology

**DOI:** 10.1016/j.gpb.2023.10.003

**Published:** 2023-10-24

**Authors:** Jun Yu

**Affiliations:** Beijing Institute of Genomics, Chinese Academy of Sciences and China National Center for Bioinformation, Beijing 100101, China

*Genomics, Proteomics* & *Bioinformatics* (GPB) is now 20 years old. It has been nurtured by an entire generation of scientists, especially genomicists and bioinformaticians in China and elsewhere, and it has certainly been blooming, from struggling to be covered by abstracting and indexing databases and drawing authorship nationwide and worldwide to fill up each issue, to defending the seemingly outdated name along with the immense increase of scientific journals in number within our own and related fields. GPB is nevertheless still thriving, benchmarked by its increasing popularity among its pertinent research communities and increasing submissions; it claims that most of the difficult episodes for startup journals are long gone and that new priorities for the journal’s prosperity are to be considered for the next 10 years or more. As to what research directions to be encouraged and publishing scopes to be entertained by GPB in the future, two issues are of importance for in-depth discussion. One concerns whether to unite more-ever-omics by looking into the future or to focus on a few of representative omics as GPB has been cultivating in the past two decades, and the other has something to do with where the actions are and what is to be expected from them in the next few decades.

The right choice is observable. GPB has been ready to morph at the beginning [1], from the “*Three Kingdoms*” to a “*United Entity*”. To pave the way for such unity, GPB has called for the second modern synthesis and new paradigms for the leading-edge research of genomics and biology [2], followed by an example of “two-track biology” [3] to stimulate a rethinking of a genuine scope for epigenetics. The new synthesis leads to a new paradigm for systems biology — holovivology (通活学) [4], [5], in which holo- (全; 统; 通) emphasizes inclusiveness, and being thorough or vivo- (生; 活) indicates physiological dimensions and their complex observational and molecular parameters in cellular details. Holovivology partitions real-time biology into five tracks or theoretic frameworks: informational, operational, homeostatic, compartmental, and plastic. In such a layout, not only the more-ever-omics ideas are to be placed in one of the five tracks, but also genetic and epigenetic mechanisms are legitimately alienated into the informational and the rest four tracks, respectively. Furthermore, it makes Albert Einstein’s eminent assertion — “*We cannot solve our problems with the same thinking we used when we created them*” — becomes testable if experimental data and theoretical models can come together to settle mechanistic issues raised in one or more of the five tracks. Two historic examples are how splicing machineries of multicellular organisms govern the gene and genome structures between plant and animal genomes and why the absolute length partition between gene-coding and non-coding sequences within a genome allocates energy utilization during replication and transcription between plant and animal genomes [5], [6], [7]. There are certainly more rules as essential as these two to be discovered in the new paradigm and the years to come.

What to do next is also crystal clear. First, a new era after the Human Genome Project came some 10 years ago known as the Precision Medicine Era [8], [9], and programs as well as legislation processes to sequence individual human genomes had both been initiated subsequently. It will not be a surprise at all, within a decade or so, that billions of personal genomes with high sequence coverage are ready for multi-dimensional scrutinization and realization in holovivological terms for clues to disease-causing variations and targets for diagnostics and therapeutics. It is predicted that such an era will be expanded into another more inclusive one immediately — the 3D Era (digital gene, digital medicine, and digital health) [10], [11]. Second, a massive effort to annotate genome sequences has to be accompanied, as well as standardized in layman languages, which must include but not be limited to: (1) all genes and their names that are comprehensive and efficacious, which are to be shared in the future by all languages aside from English; (2) adequate representative reference genomes to ensure effective comparison among populations and their subgroupings; (3) association studies between genotypes and phenotypes or across holovivotypes among the five tracks; and (4) information hubs for collecting and sharing 3D data and knowledge, which are essentially technological platforms for nationwide healthcare services. Third, novel technologies and methodologies, together with their derived tools, will come as gigantic waves that not only replace most of the outdated hardwire but also some of its scientifically flawed data. It is predictable that a fundamental breakthrough of the single-cell-single-molecule era of technology development is direct RNA sequencing; it has been funded worldwide since 2008 and will provide sequence information for modified nucleotides in RNAs at per-site-per-single-molecule resolution. GPB is determined to stand by these visions, to encourage engagements along their trajectories, and to provide an approachable podium for sharing successful stories from the valiant pioneers who enjoy working at the competitive frontiers of basic research, technology development, and real-world applications.

Although whether or not and when GPB will be ready to finish romancing its “*Three Kingdoms*” are subjected to a decision made by its editorial board and governing stakeholders in the future, we firmly believe, as what we stated 20 years ago, this celebrated quote: “*Domains under heaven, after a long period of division, tends to unite; after a long period of union, tends to divide. This has been so since antiquity*.” For the potential of holovivology, we are more than happy to agree with Max Plank: “*A new scientific truth does not triumph by convincing its opponents and making them see the light, but rather because its opponents eventually die, and a new generation grows up that is familiar with it*.”

## Competing interests

The author has declared no competing interests.

## CRediT authorship contribution statement

**Jun Yu:** Conceptualization, Writing – original draft, Writing – review & editing. The author has read and approved the final manuscript.
